# Supplementary figures and images for: The parasite Schistocephalus solidus secretes proteins with putative host manipulation functions
Source: Parasit Vectors. 2021 Aug 28;14:436. doi: 10.1186/s13071-021-04933-w (PMC8400842; doi:10.1186/s13071-021-04933-w)

## a Proteome of *S. solidus*

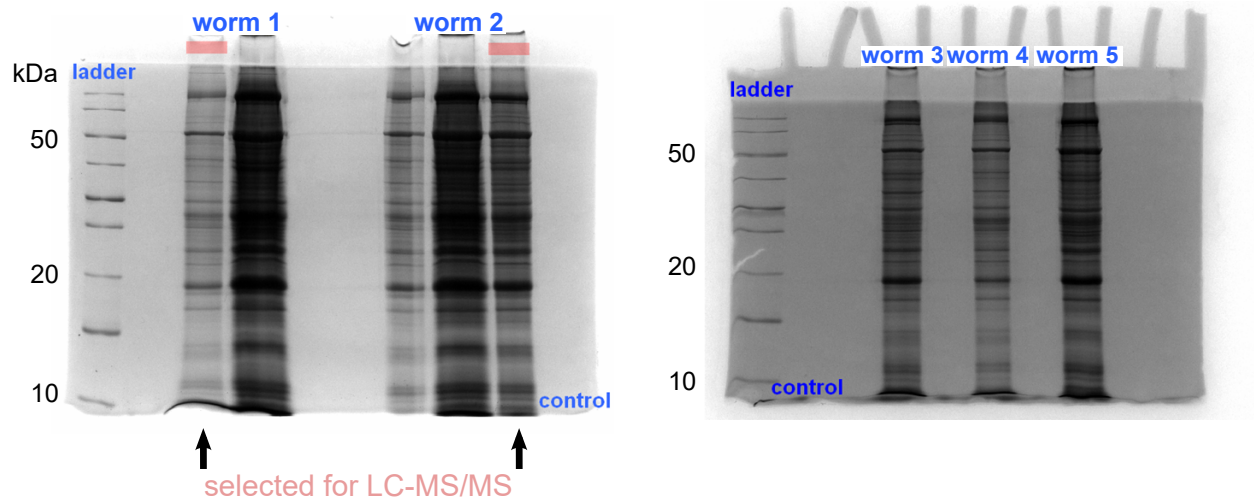

## b Secretome of *S. solidus*

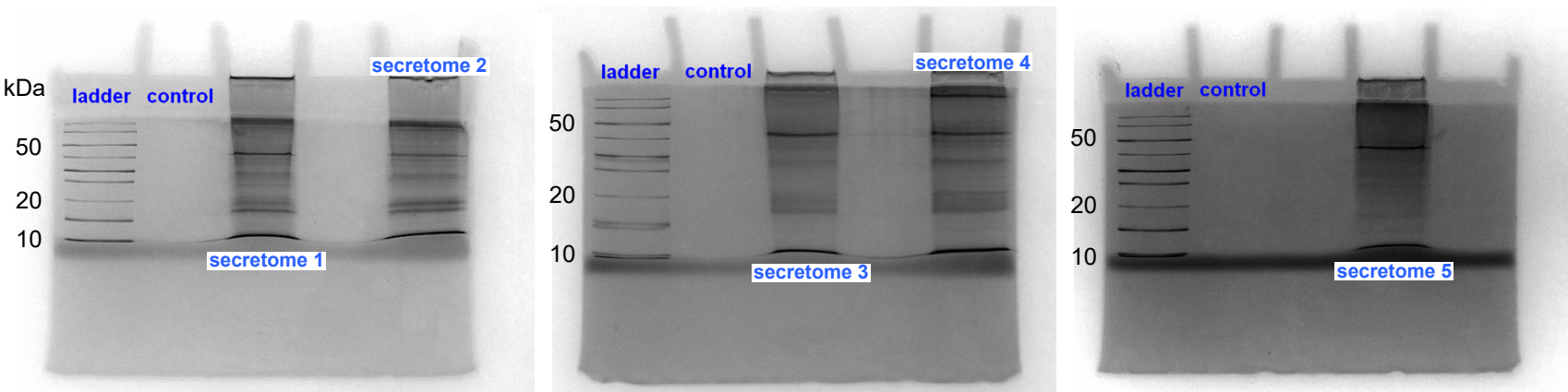

Supplement: Supplementary file 1 — Additional file 1. Visualization on SDS-PAGE gels of the protein content of the proteome and of the secretome for 5 S. solidus worms. (a) Proteome (i.e. tissues) extracts from 5 distinct worms. Note that for worm 1 and worm 2, the sample was loaded on gel either in duplicate or triplicate but only one lane was used for LC–MS/MS. (b) Secretome extracts from 5 distinct worms. On each gel, a BenchMark protein ladder (Invitrogen) and a negative control (SDS-PAGE loading buffer and water) were also loaded. [file 13071_2021_4933_MOESM1_ESM.pdf]
